# Endothelial microparticle-associated protein disulfide isomerase increases platelet activation in diabetic coronary heart disease

**DOI:** 10.18632/aging.203316

**Published:** 2021-07-20

**Authors:** Xiao-Di Sun, Lu Han, Hong-Tao Lan, Ran-Ran Qin, Ming Song, Wei Zhang, Ming Zhong, Zhi-Hao Wang

**Affiliations:** 1 The Key Laboratory of Cardiovascular Remodeling and Function Research, Chinese Ministry of Education, Chinese National Health Commission and Chinese Academy of Medical Sciences, The State and Shandong Province Joint Key Laboratory of Translational Cardiovascular Medicine, Department of Cardiology, Qilu Hospital, Cheeloo College of Medicine, Shandong University, Jinan 250012, Shandong, China; 2 Department of Geriatric Medicine, The Second Hospital, Cheeloo College of Medicine, Shandong University, Jinan 250012, Shandong, China; 3 Department of General Practice, Qilu Hospital, Cheeloo College of Medicine, Shandong University, Jinan 250012, Shandong, China; 4 Department of Geriatric Medicine, Qilu Hospital, Cheeloo College of Medicine, Shandong University, Shandong key Laboratory of Cardiovascular Proteomics, Jinan 250012, Shandong, China; 5 Department of Cardiology, The Affiliated Cardiovascular Hospital of Qingdao University, Qingdao 266071, Shandong, China

**Keywords:** protein disulfide isomerase, endothelial microparticle, platelet activation, GPIIb/IIIa, diabetes

## Abstract

Background: Endothelial microparticles (EMPs) carrying the protein disulfide isomerase (PDI) might play a key role in promoting platelet activation in diabetes. This study aimed to examine the activation of platelets, the amounts of MPs, PMPs, and EMPs, and the concentration and activity of PDI in patients with diabetic coronary heart disease (CHD) and non-diabetic CHD.

Methods: Patients with CHD (n=223) were divided as non-diabetic CHD (n=121) and diabetic CHD (n=102). Platelet activation biomarkers, circulating microparticles (MPs), the concentration of protein disulfide isomerase (PDI), and MP-PDI activity were determined. The effect of EMPs on platelet activation was investigated *in vitro*. Allosteric GIIb/IIIa receptors that bind to PDI were detected by a proximity ligation assay (PLA).

Results: Platelet activation, platelet-leukocyte aggregates, circulating MPs, EMPs, PDI, and MP-PDI activity in the diabetic CHD group were significantly higher than in the non-diabetic CHD group (*P*<0.05). Diabetes (*P*=0.006) and heart rate <60 bpm (*P*=0.047) were associated with elevated EMPs. EMPs from diabetes increased CD62p on the surface of the platelets compared with the controls (*P<*0.01), which could be inhibited by the PDI inhibitor RL90 (*P<*0.05). PLA detected the allosteric GIIb/IIIa receptors caused by EMP-PDI, which was also inhibited by RL90.

Conclusions: In diabetic patients with CHD, platelet activation was significantly high. Diabetes and heart rate <60 bpm were associated with elevated EMPs and simultaneously increased PDI activity on EMP, activating platelets through the allosteric GPIIb/IIIa receptors.

## INTRODUCTION

Cardiovascular disease (CVD) is one of the most important causes of death and disease burden worldwide. Diabetes is a major risk factor for CVD [1, 2]. The vast majority of acute cardiovascular events are associated with thrombosis, during which the activation of platelets is the triggering factor. Intensive antiplatelet therapy is a major beneficial factor for patients with diabetic coronary heart disease (CHD) [3]. However, improvements in antithrombotic therapy and reperfusion strategies can only partially contribute to outcomes in patients with diabetic CHD compared to patients with non-diabetic CHD [4]. Even with similar dual antiplatelet therapy (DAT), the incidence of cardiovascular events in diabetic patients is higher than in non-diabetic patients [5–7]. It indicates an increase in platelet activation levels in patients with diabetes, but the underlying mechanism has not been fully elucidated. In addition, diabetes has an incidence on endothelial cell injury [8–11].

GPIIb/IIIa receptor activation is the last pathway for platelet activation and aggregation. Activation of the GPIIb/IIIa receptor depends on reducing disulfide bonds and changes in the spatial conformation of integrin β3 [12]. Protein disulfide isomerase (PDI) is responsible for catalyzing the oxidation, reduction, and isomerization of disulfide bonds and plays an important role in the synthesis and function of proteins [12–15]. PDI can directly act on the GPIIb/IIIa receptor [16], activate the reduction and isomerization of disulfide bonds, and promote changes in the spatial conformation of the receptor [17–19]. It has been demonstrated that platelet-derived PDI and endothelial-derived PDI are involved in platelet activation and thrombosis by modulating the activation of the GPIIb/IIIa receptor [20–22]. Nonetheless, the origin of PDI and the mechanism of platelet activation in diabetic CVDs remain unknown.

Circulating levels of microparticles (MPs) are higher in diabetic patients and play an important role in cardiovascular complications associated with diabetes [23]. It has been confirmed that platelet-derived MPs (PMPs) and endothelial cell-derived MPs (EMPs) carry PDI and are involved in the regulation of the coagulation system and platelet function [21, 24]. In diabetic patients, the content and activity of PMPs-PDI are high, which might be the main factor for excessive activation of platelets [24]. EMPs account for only 5%-15% of total plasma MPs and are involved in platelet activation and coagulation [25, 26], but the underlying mechanism remains unclear. EMPs are also used as a marker of vascular endothelial injury [27–29]. Jasuja et al. revealed that the endothelial cell-derived PDI, but not the platelet-derived PDI, was the initiating factor of platelet activation and was required for thrombosis *in vivo*, while the platelet-derived PDI contributed to high thrombosis-related PDI [22]. When activated by EMPs, the expression of PDI on the surface of PMPs increased, and the platelets could release more PMPs carrying PDI, suggesting that PMPs act as the main carrier of PDI and exert a cascade effect on platelet activation [21]. Hence, we hypothesized that damaged endothelial cells in diabetic CHD could release EMPs carrying PDI, binding to the GPIIb/IIIa receptor on the platelet surface, and participate in early platelet activation to enhance the release of PMPs carrying PDI, which further activates platelets and initiates thrombosis. It has been shown *in vitro* and *in vivo* that EMPs from diabetic patients can activate platelets in a stronger manner than EMPs from non-diabetic patients [21, 30].

The study hypothesis was that damaged endothelial cells in diabetic CHD release EMPs carrying PDI, binding to the GPIIb/IIIa receptor on the platelet surface, and participating in early platelet activation to enhance the release of PMPs carrying PDI, which further activates platelets and initiates thrombosis. In order to test this hypothesis that EMP-PDI plays a key role in promoting platelet activation in diabetes, we tested the activation of platelets, the amounts of MPs, PMPs, and EMPs, as well as concentration and activity of PDI, in patients with diabetic CHD and non-diabetic CHD, and confirmed the role of PDI in platelet activation *in vitro*. These findings further investigate the molecular mechanism of platelet activation and new perspectives and strategies for antiplatelet therapy.

## MATERIALS AND METHODS

### Study population

Between June 2018 and June 2019, patients with CHD admitted to the Cardiology Department, Qilu Hospital of Shandong University, were selected for this study. The patients were divided into two groups according to the presence or absence of concomitant diabetes. The inclusion criterion for CHD was: one or more coronary arteries had stenosis ≥50% based on coronary angiography or coronary computed tomographic angiography (CTA) results. Type 2 diabetes was diagnosed according to the Guidelines for the prevention and treatment of type 2 diabetes in China 2017 [31]: (1) clinical symptoms of diabetes, random blood sugar ≥11.1 mmol/L (200 mg/dl); (2) fasting blood sugar ≥7.0 mmol/L (126 mg/dl); and (3) underwent an oral glucose tolerance test (OGTT), with 2-h venous plasma glucose concentration ≥11.1 mmol/L. Patients who met one of the above criteria or had a history of diabetes were diagnosed with type 2 diabetes. Patients with valvular heart disease, history of malignant tumors, hematopoietic system disease, rheumatic immune system disease, severe infectious diseases, undergoing hormone replacement therapy, underwent coronary stent implantation, atrial fibrillation, acute myocardial infarction, and grade III-IV cardiac function were excluded.

### Clinical data

Basic clinical data included gender, age, waist-to-hip ratio, body mass index (BMI), hypertension, hyperlipidemia, stroke, smoking, drinking, medication, and family history. The patients' basic clinical data, including gender, age, history of hypertension, history of hyperlipidemia, history of stroke, history of smoking, history of drinking, history of medication, and family history, were obtained by interviews. Waist-to-hip ratio and BMI were acquired from measuring the waist, hip, height, and weight.

### Specimen collection

The patients were newly admitted. They were taking their usual drugs as they received at home since they had underlying concomitant diseases (DM, hypertension, or CHD). After fasting overnight for 12-14 h, 16 ml of blood was drawn from the cubital vein of the patients, of which 8 ml was coagulated, 4 ml was sent to our laboratory for determination of serum biochemical indicators, and the remaining 4 ml was centrifuged at 1000 g for 20 min, followed by separation of serum and cryopreserved at -80° C for determination of PDI concentration. We used 2 ml of blood with EDTA to determine blood routine indicators. From the 5.4 ml of blood with 0.109 mM trisodium citrate dihydrate, 200 μl of whole blood was used for the determination of CD62p expression on platelet membrane, GPIIb/IIIa expression on platelets, as well as the percentage of platelet leukocyte aggregates using flow cytometry, all of which were completed within 20 min after blood collection. Another 2 ml was centrifuged at 3000 g for 10 min, centrifuged at 20,500 g for 60 min, followed by separation of MPs and cryopreserved at -80° C for detection of activity of MP PDI enzyme. The remaining 3 ml was centrifuged twice at 1500 g for 15 min, then centrifuged at 12,000 g for 3 min, followed by separation of platelet-free plasma, and cryopreserved at -80° C for the detection of MPs.

To minimize the effect of storage time of the specimen on platelet activation, the antibody marking of platelet activation biomarkers including CD62p, PAC-1, PLA, PMA, PNA, and PLyA for flow cytometry were all completed within 20 minutes of blood sampling and were tested immediately. The patients were tested on the day he/she was enrolled, so not all the samples were assayed at the same time. The number of MPs, EMPs, and PMPs and the serum levels of PDI and MP-MDI were assayed from the frozen specimens (-80° C) and were all assayed at the same time.

### The activated GPIIb/IIIa and CD62p on the platelet surface in whole blood was detected by three-color flow cytometry

BD flow cytometry tubes were labeled, and negative control tubes and experimental tubes were prepared for fluorescent antibody staining. CD61 PerCP 5 μL (Biolegend), PAC-1 FITC 10 μl (BD Biosciences), and CD62p PE 10 μl (BD Biosciences) were added to the experimental tubes. The appropriate volume of isotype control and RGDS 10 μl were added to the negative control tubes. Then, 5 μL of unstimulated or activated fresh whole blood was added within 10 minutes of blood collection. An appropriate amount of PBS was added to make up the final volume of 100 μL. The tubes were incubated for 20 minutes at room temperature in the dark. Then, 1 mL of cold (2° to 8° C) 1% paraformaldehyde solution was added to each tube and vortexed. The fixed cells at 2° to 8° C were incubated in the dark for at least 30 minutes. Then the samples were subjected to flow cytometry (BD FACS Caliber, BD, USA).

### Platelet leukocyte aggregates were detected by flow cytometry

BD flow cytometry tubes were labeled, and negative control tubes and experimental tubes were prepared for fluorescent antibody staining. FITC CD45 5 μL (BD Biosciences) and APC CD41a (BD Biosciences) were added to the experimental tubes. An appropriate volume of isotype control was added to the negative control tubes. Then, 50 μL of unstimulated or activated fresh whole blood was added within 10 minutes of blood collection, with an appropriate amount of PBS to make up the final volume of 100 μL. The tubes were incubated for 20 minutes at room temperature in the dark. Then, 1 ml of lysing solution (Becton, Dickinson, and Co.) was added to each tube and vortexed for 10 minutes and centrifuged at 1300 rpm for 5 minutes. The supernatant was discarded, and 500 μL of 1% paraformaldehyde was added. Then, the samples were subjected to flow cytometry (BD FACS Caliber, BD, USA).

### Circulating MP measurement

MPs were measured by flow cytometry. MPs selection was based on particle size, presence of a common surface marker (phosphatidylserine), and specific surface antigens according to the cell origin. Each PFP (50 μl) was incubated with appropriate monoclonal antibodies (mAbs) in 100 μl of filtered Annexin-V Binding Buffer (BD Biosciences) for 45 minutes at room temperature in the dark. Then, 50 μl of ACBP-20-10 particles (Spherotech) was added. The sample was run in the flow cytometer (CytoFLEX) to obtain the number of events for the particles and the samples. In the first step, the MPs isolated from the plasma were gated (R1) based on their forward (FSC) and violet side (VSSC) scatter distribution compared to the distribution of synthetic 0.1–1.0 μm Megamix-Plus FSC Beads (BioCytex) for cytometer settings in MP analysis. After that, events present in R1 were accessed for their positive staining for Annexin V (BD), which binds to phosphatidylserine. Finally, Annexin V+ events were gated with conjugated mAbs against the cell markers CD41a-PECy5 (platelets), CD144-PE (endothelial cells), CD14-Bv510 (monocytes), CD235a-Pevio770 (erythrocytes). Data analysis was performed using Kaluza Analysis (version 2.1).

### Platelet preparation

Blood was drawn from a median cubital vein to anticoagulation tubes with 0.109 mol/L sodium citrate after fasting overnight for 12-14 hours. The blood samples were centrifuged at 120 g for 10 minutes at room temperature, and platelet-rich plasma (PRP) was obtained. The PRP was centrifuged at 800 g for 10 minutes to precipitate platelets. The platelets were resuspended, and their concentration was adjusted to 1×10^7^/ml using BD Trucount^TM^ tubes, then used at once in experiments.

### Separation and identification of EMPs

Blood (3 ml) with 0.109 mM trisodium citrate dehydrate was centrifuged at 3000 g for 10 min, centrifuged at 20,500 g for 60 min, followed by the separation of MPs. Subsequently, 70 μl of buffer and 10 μl of CD41/CD61-PE were added to the MPs and mixed. The mixture was incubated at room temperature for 30 minutes away from light. Then, 20 μl of PE-labeled immunomagnetic beads were added and mixed. The mixture was incubated at room temperature for 2 hours away from light. An LS column was placed in a magnetic field. The magnetic sorting buffer (0.5 ml) was added and allowed to flow through by gravity to prepare the column. The incubated supernatant was loaded onto the sorting column. A magnetic sorting buffer (500 μL) was added to the column and allowed to flow through gravity. This step was repeated three times to collect the unlabeled eluent. The liquid was centrifuged at 20,500 g for 60 minutes, and the pellet was resuspended in 80 μl of buffer. Then, 20 μl of CD144 MicroBeads was added to the MPs. The tubes were mixed well and incubated overnight in the refrigerator (2-8° C). The above magnetic separation steps were repeated to collect the labeled eluent. The amount of EMP was adjusted to 2×10^7^/ml by flow cytometry. Subsequently, 5 μl of CD144-PE antibody (BD Bioscience) and 5 μl of anti-mouse PDI-FITC antibody (Santa Cruz) were added in a BD tube, followed by 10 μl of EMP suspension. PBS was added to a final volume of 100 μl of the reaction mixture. The mixture was incubated at 37° C for 20 minutes away from light. Finally, 200 μl of 4% paraformaldehyde was added. The samples were mixed and immediately subjected to flow cytometry. The structure of EMP was observed by TEM. The qNano particle analyzer was used to determine the average diameter of EMPs.

### Activation of platelets by EMPs measured by flow cytometry

Platelets were processed in accordance with the procedures mentioned above. The platelets (1×10^7^/ml) were divided into three groups: control group, EMP (2×10^7^/ml) group, and ADP (10 μg/ml) group, and were incubated at 37° C for 30 minutes. After that, 20 μl of platelet suspension was added to a BD tube, along with 5 μl of PE CD62p (BD Biosciences) and 75 μl of PBS, and mixed gently. Simultaneously, the isotype control was prepared. All samples were incubated at room temperature in the dark for 30 minutes, fixed with 200 μL of 1% paraformaldehyde, mixed, and immediately mounted for testing. After booting and calibration, the forward scatter angle, lateral scatter angle, and the fluorescence detection signal of the flow cytometer was set to logarithmic amplification mode to measure 20,000 platelets. Data analysis was performed using Kaluza Analysis (version 2.1).

### PDI activity by insulin transhydrogenase assay and ELISA detection of PDI concentration

PDI activity was determined using the insulin transhydrogenase assay. Insulin (250 μl. 1 mg/mL), MPs (50 μl), and DTT (10 μL, 100 mM) was added to a 96-wells plate and mixed gently. Detect absorbance at 650 nm was measured at once and every 10 minutes by a microplate reader for 90-120 minutes. The PDI ELISA kit (SEB061Hu) was used to detect the serum PDI concentration, and the operation was performed according to the instructions. The human PDI ELISA kits were purchased from Cloud-Clone Crop.

### Effects of PDI inhibitors on EMPs-mediated platelet activation by flow cytometry

We pretreated the EMPs (2×10^7^/ml) with an anti-PDI monoclonal antibody (RL90, 5 μg/ml) and IgG (5 μg/ml) at 37° C for 30 minutes. Platelets from healthy individuals (staff working with the research team) were collected to simply observe the effect of EMP on platelet activation, avoiding the effects of other factors such as hypertension. The healthy volunteers (n=11) were 25-30 years of age and without hypertension, coronary heart disease, or diabetes. After fasting overnight for about 12-14 hours, elbow venous blood samples were taken (0.109 mM sodium citrate for anticoagulation) the next morning. After centrifugation at 120 g for 10 minutes, the supernatant (PRP) was collected, and then 100 nmol/L PGE1 was added. After centrifugation at 800 g for 10 minutes, the supernatant was discarded. The platelet pellet was washed and resuspended in modified tyrode solution (137 mmol/L NaCl, 2.7 mmol/L KCl, 12 mmol/L NaHCO_3_, 0.4 mmol/L NaH_2_PO_4_, 5 mmol/L HEPES, 0.1% glucose, 0.35% bovine serum albumin, and 100 nmol/L PGE1, pH 7.2). The cell count was adjusted to 1×10^7^/ml by the flow type fluorescent microsphere absolute counting method. Then, the platelets of healthy people were stimulated by RL90 (PDI-inhibitor) and EMPs pre-treated by the same IgG type. CD62p, the biomarker of platelet activation, was tested by flow cytometry. The samples were subjected to flow cytometry to detect the expression of the platelet activation marker P-selectin.

### The binding of EMPs-PDI to the GP IIb/IIIa receptor on the surface of platelets was confirmed using the Duolink® *in-situ* proximity ligation assay (PLA)

We decided to monitor the EMPs-PDI and GPIIb/IIIa interaction by PLA. The principle of this assay was based on the staining of PDI and GPIIb/IIIa proteins by two antibodies, which were next revealed by secondary antibodies conjugated with oligonucleotides. The platelets and EMPs were isolated. The samples were incubated with antibodies and PLA probes, followed by ligation and amplification according to the experimental protocol. In the presence of hybridization solution and ligase, the two oligonucleotides formed with PLA a circle in case of the proximity of the proteins, i.e., PDI and GPIIb/IIIa. Then, polymerase and nucleotides formed the rolling circle amplification, which was visualized in red fluorescence. It indicated that the red dotted particles observed by immunofluorescence microscopy represent activated GP IIb/IIIa receptor binding to PDI. Thus, even though the EMPs were mixed with platelets later, the platelets were never directly exposed to binding to the PDI antibodies. Therefore, the fluorescent signal that eventually formed was considered to be the binding of EMP-PDI and platelet-α2bβ3.

### Statistical analysis

Continuous data were presented as means ± standard deviations (normal distribution according to the Kolmogorov-Smirnov test) or median (P25, P75). Differences between the two groups were analyzed using Student's t-test for independent samples, and differences among multiple groups were analyzed using a one-way analysis of variance (ANOVA) with the LSD post hoc test. The Mann-Whitney U-test was used for non-normally distributed data. Multiple linear regression (enter) was used to identify the factors associated with EMPs in CHD patients, the diabetic CHD group, and the non-diabetic CHD group, respectively. Statistical graphs were processed using the GraphPad Prism V6.01 software package. Differences were considered statistically significant at P<0.05. Analyses were performed using SPSS 24.0 (IBM, Armonk, NY, USA).

The sample size was calculated based on the results of a preliminary study, in which the non-diabetic CHD group and diabetic CHD group included 11 and 10 patients, respectively. A post hoc power analysis was performed. The sample size calculation was carried out according to CD62p, Pac-1, PLA, PNA, PMA, PLyA, MPs, EMPs, and PMPs. Using α=0.05, β=0.10, ratio of 1:1 (k=1), power of 90%, the actual difference values (δ) and standard deviation (σ) between the two groups for each tested variable (but keeping the one with the largest required sample size), 92 patients were necessary for each group, for a total of 184. Since the non-diabetic group had 121 patients and the diabetic group had 102, the sample size was sufficient. The formula was:

$$n_{2}=\frac{{\left(z_{1-\frac{\alpha }{2}}+z_{1-\beta }\right)}^{2}\sigma^{2}(1+\frac{1}{k})}{\delta^{2}}$$

### Study approval

This study was approved by the Ethics Committee of Qilu Hospital of Shandong University (No. KYLL-2014(KS)-079). All patients signed the written informed consent form.

### Data availability statement

The datasets used and/or analyzed during the present study are available from the corresponding author on reasonable request.

## RESULTS

### Baseline data

A total of 223 patients were enrolled. The non-diabetic CHD group consisted of 121 patients, with a mean age of 60.37±9.50 years, including 84 men (69.42%) and 37 women (30.58%). The diabetic CHD group consisted of 102 patients, with a mean age of 62.70±8.60 years, including 62 men (60.78%) and 40 women (39.22%). The clinical and biological characteristics of the patients with non-diabetic and diabetic CHD are shown in Supplementary Table 1. There were no significant differences in age (*P*=0.059), gender (*P*=0.177), aspirin use (*P*=0.462), ADP receptor inhibitor use (*P*=0.055), and statin use (*P*=0.935) between the two groups. Meanwhile, hypertension, hyperlipidemia, family history of diabetes, calcium channel blockers (CCB) drug use rate, systolic blood pressure, waist-to-hip ratio, fasting blood glucose, fibrinogen, and Gensini score were higher in the diabetic CHD group than in the non-diabetic CHD group. Moreover, hemoglobin levels, high-density lipoprotein cholesterol, and chloride ions were lower in the non-diabetic CHD group.

### Significantly higher platelet activation and platelet-leukocyte aggregate levels in the diabetic CHD group as compared to the non-diabetic CHD group

**Figure 1:**
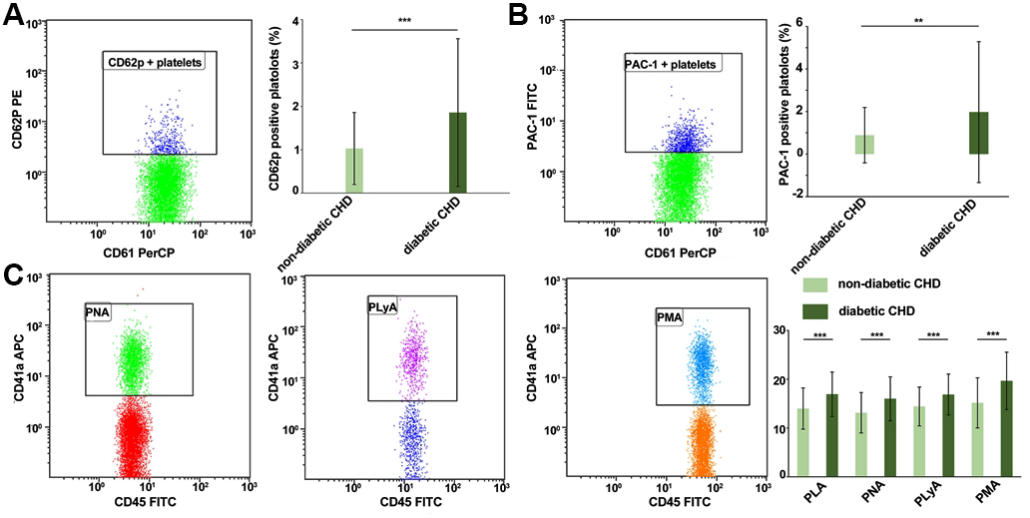
**Comparison of platelet activation and platelet-leukocyte aggregate levels in the diabetic CHD group and non-diabetic CHD group.** (**A**) Detection of CD62p expression level on platelet surface by flow cytometry of diabetic CHD group and non-diabetic CHD group. (**B**) Detection of PAC-1 expression level on platelet surface by flow cytometry of diabetic CHD group and non-diabetic CHD group. (**C**) Detection of platelet-leukocyte (CD45+ CD41a+) adhesion by flow cytometry of diabetic CHD group (n=121) and non-diabetic CHD group (n=102). Values are expressed as mean±SD. Analyses were done by t-test for independent samples. *P<0.05; **P<0.01; ***P<0.001 vs. diabetic CHD group. Definition of abbreviations: PLA: platelet-leukocyte aggregate levels; PMA: platelet monocyte aggregate levels; PLyA: platelet lymphocyte aggregate levels; PNA: platelet neutrophil aggregate levels.

Compared with the non-diabetic CHD group, the expression levels of CD62p (1.03%±0.83% vs. 1.86%±1.71%, *P*<0.001, Figure 1A) and PAC-1 (0.89%±1.31% vs. 1.97%±3.33%, *P*<0.01, Figure 1B) on the platelet surface, as well as the proportions of PLA (13.98%±4.24% vs. 16.91%±4.59%, *P*<0.001), PNA (13.12%±4.16% vs. 15.99%±4.53%, *P*<0.001), PLyA (14.41%±3.99% vs. 16.85%±4.21%, *P*<0.001), and PMA (15.15%±5.17% vs. 19.65%±5.92%, *P*<0.001, Figure 1C) were significantly higher in the diabetic CHD group, which indicated higher platelet activation in the diabetic CHD group compared with the non-diabetic CHD group.

### Significantly higher circulating MPs, EMPs, PDI, and activity of MP-PDI in the diabetic CHD group compared with the non-diabetic CHD group

**Figure 2:**
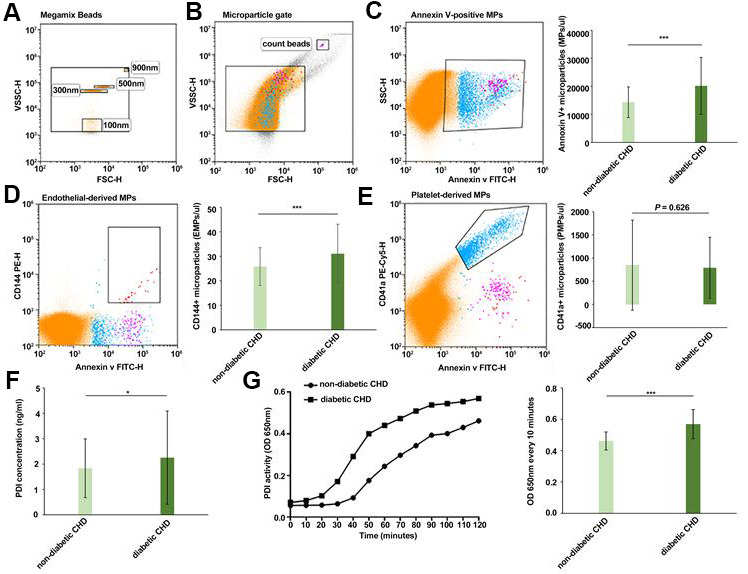
**Comparison of circulating MPs, EMPs, PDI, and activity of MP-PDI in the diabetic CHD group and non-diabetic CHD group.** (**A**) Megamix Beads distribution (0.1 μm/0.3 μm /0.5 μm /0.9 μm). (**B**) MPs gate and count beads gate. (**C**) Circulating Annexin V+ MPs in non-diabetic CHD group and diabetic CHD group. (**D**) Circulating EMPs (Annexin V+/CD144+) in the non-diabetic CHD group and diabetic CHD group. (**E**) Circulating PMPs (Annexin V+/CD41+) MPs in non-diabetic CHD group and diabetic CHD group. (**F**) Detection of PDI concentration in serum of non-diabetic CHD group and diabetic CHD group. (**G**) Detection of PDI activity in MPs by insulin transhydrogenase assay of non-diabetic CHD group (n=121) and diabetic CHD group (n=102). Values are expressed as mean±SD. Analyses were done by t-test for independent samples. *P<0.05; **P<0.01; ***P<0.001 vs. non-diabetic CHD group.

Flow cytometry was used to measure the number of MPs in platelet-deficient plasma in both groups. The results showed that the total number of MPs (14,330.10±5510.25 vs. 20,189.81±10,206.76 MPs/μl, *P*<0.001, Figure 2C) as well as the number of EMPs (25.82±7.75 vs. 31.07±12.12 EMPs/μl, *P*<0.001, Figure 2D) were significantly higher in the diabetic CHD group compared with the non-diabetic CHD group, while the number of PMPs (846.17±975.80 vs. 790.56±663.98 PMPs/μl, *P*=0.626, Figure 2E) showed no significant difference between the two groups.

ELISA was used to determine the serum PDI concentration. The results revealed that the serum PDI concentration (1.83±1.16 vs. 2.25±1.84 ng/ml, *P*<0.05, Figure 2F) in the diabetic CHD group was higher than in the non-diabetic CHD group. Insulin transhydrogenase assay was used to detect PDI activity in MPs. It was found that the PDI activity (0.46±0.06 vs. 0.57±0.09 OD_650 nm_, *P*<0.001, Figure 2G) was higher in the MPs of patients with diabetic CHD compared with the non-diabetic CHD group.

### Factors associated with higher EMPs

To screen for factors influencing EMPs, a multivariable linear stepwise regression was performed. The results showed that diabetes (*P*=0.006) and HR <60 bpm (*P*=0.047) were associated with high EMPs (Supplementary Table 2). In the diabetic CHD group, a multivariable linear stepwise regression was also performed, demonstrating that heart rate <60 bpm (*P*=0.037) was associated with high EMPs (Supplementary Table 3). In the non-diabetic CHD group, the multivariable linear stepwise regression did not show any significant factors associated with high EMPs (Supplementary Table 4).

### EMPs from diabetic CHD activate platelets

**Figure 3:**
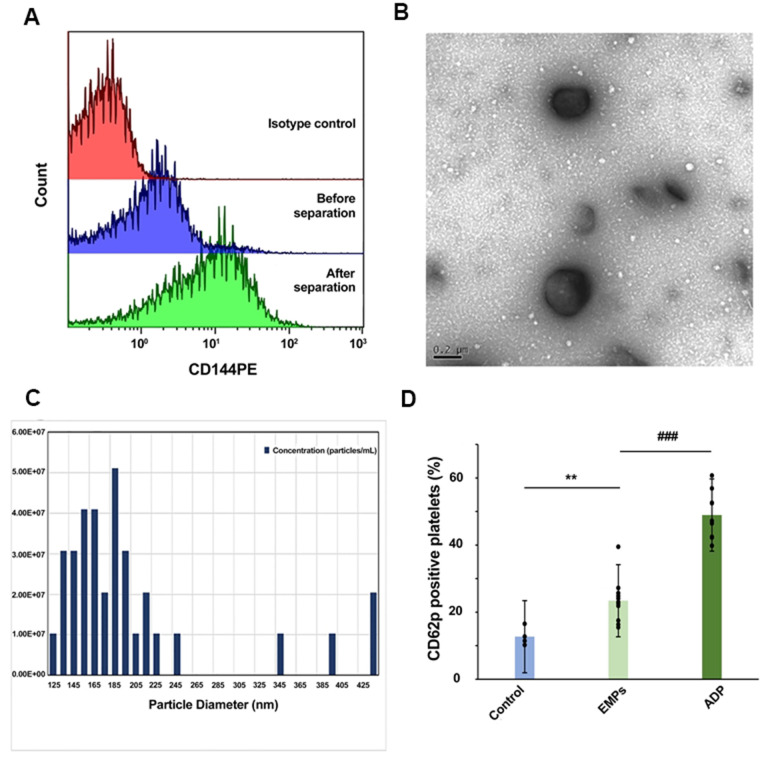
**Isolation and identification of EMPs and EMPs mediated platelet activation.** (**A**) The positive rate of CD144 in the plasma before and after isolation. (**B**) Transmission electron microscopy was used to observe the obtained microparticles with a diameter >100 nm. (**C**) Quantification and size estimation of EMPs by qNANO. (**D**) Adenosine diphosphate (ADP) (10 μg/ml), EMPs (2×10^7^/ml), and an equal amount of PBS were used to stimulate the platelets of healthy people. Detect platelet expression of CD62p by flow cytometry. Histograms represent the CD62p% change. **P<0.01 *vs.* control, ###*P*<0.001 *vs.* EMPs treatment (n=4-11). Data are analyzed by one-way ANOVA.

EMPs were first isolated using gradient density centrifugation and immunocapture techniques. The positive rate of CD144+ EMPs was increased from 20% before isolation to 76% after isolation (Figure 3A). Then, the obtained EMPs were observed by transmission electron microscopy, which showed double-membrane follicles with a diameter >100 nm (Figure 3B). Subsequently, the qNano system was used to monitor the concentration and size of EMPs (Figure 3C). Three groups were set up: control group, EMP stimulation group, and ADP-positive control group, which were incubated with platelets collected from normal people for 30 min, followed by detecting CD62p expression on the surface of platelets by flow cytometry. The results showed that CD62p expression was higher in the EMP stimulation group (23.42%±6.58% vs. 12.70%±2.73%, *P*<0.01) and ADP-positive control group (48.95%±7.14% vs. 12.70%±2.73%, *P*<0.001) compared with the control group (Figure 3D).

### EMPs carrying PDI activate platelet GPIIb/IIIa receptors

**Figure 4:**
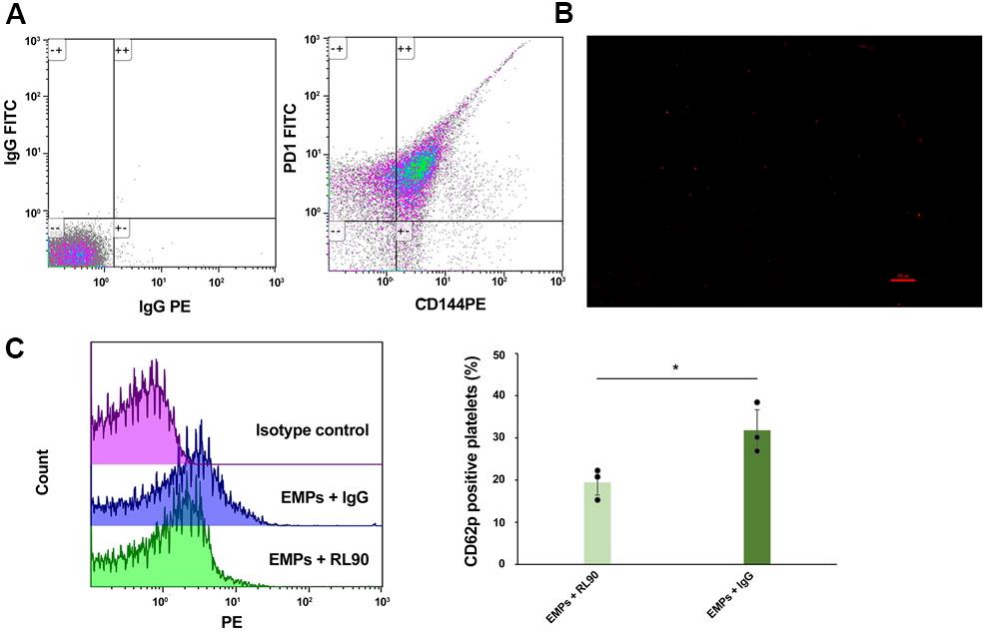
**EMPs-mediated PDI-dependent platelet activation: PDI was carried by EMPs, and PDI inhibitor (RL90) inhibit EMPs-mediated platelet activation.** (**A**) Bicolor flow cytometry confirmed EMPs carrying PDI. (**B**) The Duolink® *in-situ* proximity ligation assay was used to detect the direct binding of EMP-PDI and GPIIb/IIIa receptors on the platelet surface. The red dot granule represents PDI binding to GPIIb/IIIa receptor (scale = 100 μm). (**C**) Comparisons of the CD62p expression level on the platelet surface of EMPs+RL90 and EMPs+IgG. Values are expressed as mean±SD. Analyses were done by t-test for independent samples. *P<0.05 vs. EMPs+RL90 (n=3).

We confirmed that PDI was present on EMPs by CD144 PE and PDI FITC double-color flow cytometry (Figure 4A). Then, the PLA was used to detect the interaction between the platelet GPIIb/IIIa receptors and EMP-PDI (Figure 4B). The red-spotted MPs observed by immunofluorescence microscopy represented the allosteric GIIb/IIIa receptors that bind to PDI. After that, the platelets collected from healthy people were stimulated with EMPs separately incubated with PDI inhibitor RL90 and IgG (isotype control of RL90) for 30 minutes, followed by detecting CD62p expression on the surface of platelets by flow cytometry. The results revealed that CD62p expression on the surface of platelets in the EMPs+RL90 group was decreased compared to the EMPs+IgG group (31.81%±5.94% vs. 19.43%±3.36%, *P*<0.05, Figure 4C), which indicated that RL90 could partially inhibit the PDI pathway and attenuate EMPs-induced platelet activation.

### Subgroup analyses

A multivariable linear stepwise regression was performed (Supplementary Table 5), and a forest plot was displayed (Supplementary Figure 1). As for hypertension, CHD combined with diabetes was associated with higher EMPs in patients with hypertension, while diabetic CHD was not associated with higher EMPs in patients without hypertension. As for hyperlipidemia, CHD combined with diabetes was associated with higher EMPs in patients without hyperlipidemia, while diabetic CHD was not associated with higher EMPs in patients with hyperlipidemia. As for the use of CCB, CHD combined with diabetes was associated with higher EMPs in patients without receiving CCB, while diabetic CHD was not associated with higher EMPs in patients receiving CCB.

## DISCUSSION

In this study, platelet activation was high in diabetic CHD patients, accompanied by higher MPs, EMPs, PDI, and activity of MP-PDI. Diabetes and heart rate <60 bpm were associated with elevated EMPs. Furthermore, the elevated EMPs activated platelets by allosteric GPIIb/IIIa receptors through PDI on the surface. These results suggest that in daily diagnosis and treatment, patients at risk of CVD would benefit from anticoagulant and/or antiplatelet therapy for primary prevention of CVD.

Previous studies confirmed the presence of platelet hyper-reactivity/activation in diabetes [32]. These results have been further confirmed by the present study, especially in the case of an equivalent dose of antiplatelet drugs, and the platelets showed hyper-reactivity/activation in diabetes, which plays a central role in accelerating atherothrombosis. The leading cause of hyper-reactivity of platelets remains unclear. Nonetheless, many recent studies have shown that MP plays a key role in hemostasis and thrombosis. The procoagulant characteristics of MP have been confirmed by the increased risk of involvement in various thromboembolic events, including thalassemia [33], atrial fibrillation [34], atherosclerosis, venous thrombosis [35], and other diseases. EMPs are biomarkers of vascular endothelium that reflect the pathological process of diabetes-related vascular diseases and endothelial dysfunction [36–38]. The present study showed that the release of EMPs was higher in patients with diabetes and CHD compared with those without diabetes, which was consistent with previous reports [39, 40]. There are many coagulation factors on the surface of EMPs, such as P-selectin, phosphatidylserine, tissue factor, and coagulation factors, among others, which play important roles in platelet activation and pro-coagulation process [28]. The EMP levels can affect the severity, lesion size, and prognosis of acute ischemic stroke [41]. Therefore, EMP may be an important source of platelet activation in diabetes.

During the occurrence and development of CVDs, changes in MPs' number, composition, and function are associated with a hyper-coagulation state, inflammation, and endothelial dysfunction [42]. In this study, we revealed that diabetes and heart rate were clinical factors associated with higher EMPs. In diabetes, insulin resistance, hyperglycemia, and hyperlipidemia can induce the apoptosis of endothelial cells through multiple signaling pathways [43–45], which further leads to an increase of EMPs. It was reported that the circulating MPs were elevated in obese patients [46, 47]. However, the EMP levels decreased, and the endothelial function improved after six months of aerobic exercise [48]. It suggests that high BMI is a link between high circulating EMPs and endothelial dysfunction. Compared with young people, the concentration of EMPs in the elderly is elevated, and the release of EMPs by endothelium activation may be related to the declined vascular function associated with age [49]. Nevertheless, in the present study, multiple linear regression analyses did not show a significant association between EMPs and BMI and age. At present, no study referred to the correlation between HR and EMP level, but it was reported that central systolic and pulse pressure was higher in patients with HR ≤ 60 bpm than those with HR ≥ 80 bpm [50]. Central systolic and pulse pressure were measures of arterial stiffness, which correlate significantly with endothelial function. An increase in vessel stiffness may represent a cause of endothelial dysfunction [51]. Therefore, comprehensive treatment of diabetes and maintaining HR in a normal range might be beneficial to protect vascular endothelial function and reduce EMPs, thereby decreasing the platelet activation induced by EMPs.

The mechanism of initiation of platelet activation by EMP was further investigated. PDI is found on PMPs and EMPs and is a key enzyme for activating GPIIb/IIIa receptors to accelerate thrombosis. Nevertheless, it is unclear whether EMP-PDI is involved in the development of CVDs and the activation of platelets in diabetes. This study showed that PDI concentration and MP-PDI activity were higher in diabetic patients with CHD than non-diabetic ones. PMPs and EMPs were the main sources of MP-PDI, and there was no significant difference in PMPs between the two groups. Still, EMPs were significantly elevated in the diabetic CHD group. Hence, we considered that the high circulating PDI and PDI activity is largely attributed to the higher levels of EMP, which suggested that the process of platelet activation by EMPs was associated with PDI. To confirm this hypothesis, the platelets were incubated with EMPs, verifying that EMPs could bind to platelet GPIIb/IIIa receptors through PDI to increase CD62p expression on the surface of platelets. In contrast, PDI inhibitor RL90 could effectively reduce CD62p expression on the surface of platelets. Therefore, the amount of EMPs is high in diabetic patients, simultaneously increased PDI on EMP, which tends to activate numerous platelets by allosteric GPIIb/IIIa receptors, causing significant increases in platelet activation and the occurrence of cardiovascular events. Exploring new therapeutic methods targeting this pathway may provide new sights for the comprehensive management of diabetic CHD patients. Our previous study in diabetic mice models showed that the PDI inhibitors RL90 and rutin could partially inhibit EMP-PDI and reduce platelet activation [30]. However, we have not examined the effectiveness of rutin on EMP-PDI inhibition in clinical trials, and future prospective studies are needed to verify the results.

Obesity is known to be associated with an increased risk of coronary heart disease, peripheral artery disease, stroke, and lower limb venous thrombosis [52–54]. Obesity is associated with altered levels of clotting factors and fibrinolytic pathways that lead to hypercoagulability [52, 55, 56]. In addition, studies have reported elevated MP levels derived from different cells in obese people [57–61]. It is also reported that at 12 months after weight loss in obese patients, EMPs are decreased significantly, and the percentage of EMP reduction was significantly related to the percentage reduction in BMI [62]. Obesity is a state of chronic oxidative stress and inflammation [63]. Prolonged activation or overactivation of the endothelium by inflammatory factors can lead to endothelial dysfunction and circulation EMP detachment from the blood vessels [64]. Nevertheless, the exact relationship between obesity and EMP production remains to be confirmed. The mechanism is unknown and additional studies are necessary.

There were some limitations. Only circulating PDI and activity of total MP-PDI were explored. Perfectly, a comparison of EMP-PDI between the two groups would increase accuracy. Compared to the EMPs+IgG group, CD62p expression on the surface of platelets in the EMPs+RL90 group was decreased but not abolished. Therefore, RL90 only partially inhibits rather than completely inhibits the PDI pathway and attenuates the platelet activation induced by EMPs. In fact, the present study and the literature have no answer to this question for now. Compensatory mechanisms might play a role. Additional studies are necessary to explain this phenomenon.

## CONCLUSIONS

In diabetic CHD patients, platelet activation was significantly high. Diabetes and heart rate <60 bpm were major factors associated with higher EMPs, and simultaneously high PDI activity on EMP, which tends to activate numerous platelets by allosteric GPIIb/IIIa receptors.

## Supplementary Material

Supplementary Figure 1

Supplementary Tables
