# Supplementary material for: Endothelial microparticle-associated protein disulfide isomerase increases platelet activation in diabetic coronary heart disease
Source: Aging (Albany NY). 2021 Jul 20;13(14):18718–39. doi: 10.18632/aging.203316 (PMC8351716; doi:10.18632/aging.203316)
Supplement: Supplementary Figure 1 [file aging-13-203316-s001.pdf]

SUPPLEMENTARY FIGURE

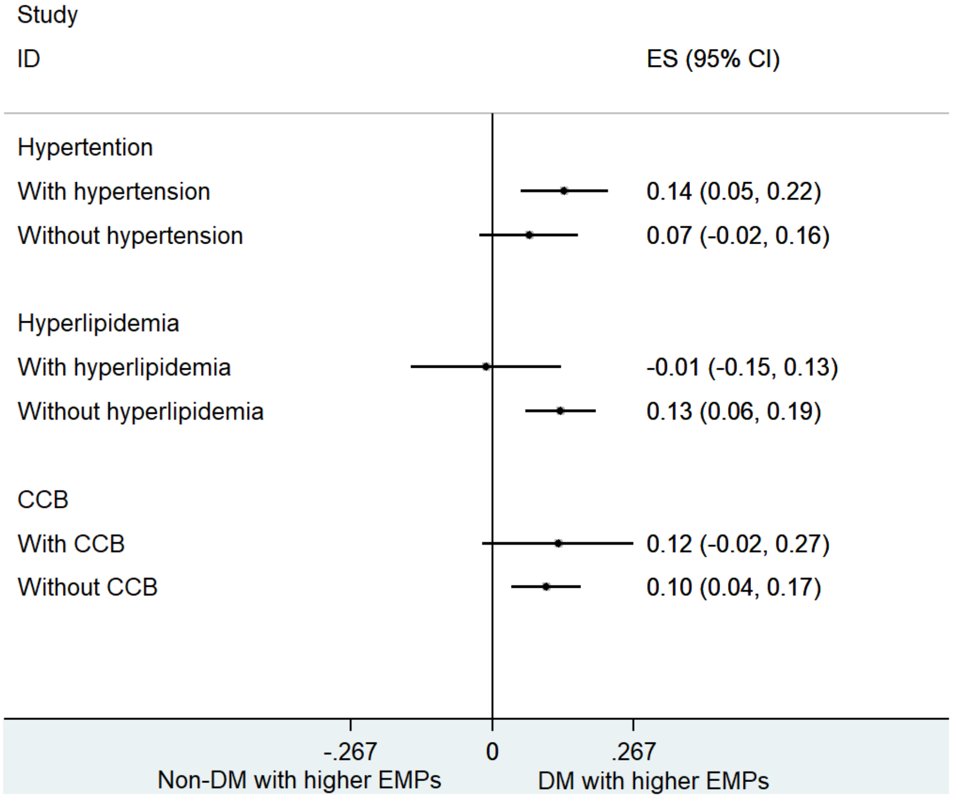

Supplementary Figure 1. Forest plot of subgroup analysis for hypertension, hyperlipidemia, and application of CCB. The figure presents the adjusted beta coefficients of diabetes in each subgroup.
