# Supplementary material for: Endothelial microparticle-associated protein disulfide isomerase increases platelet activation in diabetic coronary heart disease
Source: Aging (Albany NY). 2021 Jul 20;13(14):18718–39. doi: 10.18632/aging.203316 (PMC8351716; doi:10.18632/aging.203316)
Supplement: Supplementary Tables [file aging-13-203316-s002.pdf]

SUPPLEMENTARY TABLES

Supplementary Table 1. Clinical and biological characteristics in the study population.

Clinical features	Non-diabetic CHD (n=121)	Diabetic CHD (n=102)	*P*
Sex, Male, n (%)	84 (69.42%)	62 (60.78%)	0.177
Age (years)	60.37±9.50	62.708.60	0.059
Hypertension, n (%)	62 (51.24%)	69 (67.65%)*	0.013
Hyperlipidemia, n (%)	17 (14.17%)	27 (26.47%)*	0.020
Stroke history, n (%)	1 (0.83%)	5 (4.90%)	0.061
Smoking history, n (%)	61 (50.41%)	43 (42.16%)	0.218
Drinking history, n (%)	38 (31.40%)	32 (31.37%)	0.996
Family history of hypertension, n (%)	26 (21.49%)	19 (18.63%)	0.596
Family history of CAD, n (%)	21 (17.36%)	16 (15.69%)	0.739
Family history of DM, n (%)	2 (1.65%)	12 (11.76%) ***	0.000
Family history of CVD, n (%)	3 (2.48%)	1 (1.0%)	0.401
BMI (kg/m²)	25.69 (23.63, 27.94)	25.67 (23.83, 28.08)	0.722
WHR	0.93 (0.89, 0.97)	0.95 (0.91, 0.99)*	0.048
SBP (mmHg)	133.18±16.63	141.11±18.87**	0.001
DBP (mmHg)	77.67±12.16	75.22±10.59	0.114
TG (mmol/L)	1.30 (1.02, 1.85)	1.435 (1.11, 2.30)	0.063
TC (mmol/L)	3.96 (3.37, 4.51)	3.905 (3.22, 4.68)	0.695
HDL-C (mmol/L)	1.09 (0.93, 1.26)	1.00 (0.87, 1.17)**	0.007
LDL-C (mmol/L)	2.22 (1.88, 2.79)	2.17 (1.69, 2.76)	0.351
FPG (mmol/L)	4.74 (4.45, 5.2)	7.205 (5.96, 8.67)***	<0.001
Albumin (g/L)	44.4 (42.45, 46.15)	44.5 (42.8, 47)	0.425
UA (μmol/L)	304.5 (261, 377.5)	299 (247, 356.5)	0.361
Cr (μmol/L)	69 (61, 79)	66 (59, 79)	0.210
Na^+ (mmol/L)	143 (142, 145)	143 (141, 144)*	0.044
K+ (mmol/L)	4.02 (3.84, 4.22)	4.02 (3.82, 4.25)	0.906
Cl- (mmol/L)	106 (104, 108)	105 (103, 108)*	0.036
NT-proBNP (pg/ml)	63.78 (33.205, 142.2)	137.6 (66.645, 322.1)***	<0.001
WBC ($\times 10^9$/L)	5.635 (4.95, 6.67)	6.08 (5.42, 7.03)*	0.020
RBC ($\times 10^9$/L)	4.69±0.45	4.62±0.46	0.273
HB (g/L)	140.5 (133, 150)	137 (129, 146)*	0.024
PLT ($\times 10^9$/L)	222 (179.5, 266)	213 (185, 258)	0.640
PT (s)	11.3 (10.9, 11.9)	11.4 (10.8, 11.8)	0.869
APTT (s)	30.6 (29.2, 32.65)	30.25 (28.8, 32.1)	0.276
FIB (g/L)	2.90±0.55	3.17±0.54***	<0.001
D-dimer	0.07 (0.05, 0.135)	0.08 (0.05, 0.13)	0.340
Gensini score	27 (14, 50)	41 (25, 66.5)**	0.001
Treatment			
Aspirin, n (%)	104 (95.95%)	84 (82.35%)	0.462
ADP receptor inhibitor, n (%)	69 (57.50%)	45 (44.12%)	0.055
ACEI, n (%)	15 (12.40%)	14 (13.73%)	0.769
ARB, n (%)	26 (21.49%)	30 (29.41%)	0.174
CCB, n (%)	24 (19.83%)	46 (45.10%) ***	<0.001
β-blocker, n (%)	64 (52.89%)	64 (62.75%)	0.138
Statin, n (%)	86 (71.07%)	73 (73.74%)	0.935
Diuretics, n (%)	10 (8.26%)	9 (8.82%)	0.882

Metformin	-	56 (54.95%)
Sulfonylurea	-	19 (18.63%)
TZDs	-	4 (3.92%)
Glinide	-	7 (6.86%)
α-glucosidase inhibitor	-	29 (28.43%)
DPP-4 inhibitor	-	4 (3.92%)
Insulin	-	23 (22.55%)

Values are expressed as mean±SD, median (P25, P75), or number (%). Analyses were done by chi-square test (for categorical data) or t-test for independent samples (for continuous data). *$P<0.05$; **$P<0.01$; ***$P<0.001$ vs. diabetic CHD.

Definition of abbreviations: BMI, body mass index; WHR, waist-to-hip ratio; SBP, systolic blood pressure; DBP, diastolic blood pressure; TG, triglyceride; TC, total cholesterol; HDL-C, high-density lipoprotein cholesterol; LDL-C, low-density lipoprotein cholesterol; FPG, fasting plasma glucose; UA, uric acid; Cr, creatinine; NT-proBNP, N-terminal pro–B-type brain natriuretic peptide; WBC, white blood cell; RBC, red blood cell; PLT, platelet; HB, hemoglobin; PT, prothrombin time; APTT, activated partial thromboplastin time; FIB, fibrinogen; ACEI, angiotensin-converting enzyme inhibitor; ARB, angiotensin receptor blocker; CCB, calcium-channel blocker; TZDs, thiazolidinediones; DPP-4, dipeptidyl peptidase-4.

Supplementary Table 2. Multiple regression report in CHD patients.

Independent variable	β	*t*	*P*
Constant	2.879	23.031	<0.001
Sex	0.003	0.051	0.959
Age	-0.013	-0.445	0.657
Hypertension	0.076	1.480	0.140
Hyperlipidemia	0.055	0.858	0.392
Myocardial infarction history	-0.053	-0.205	0.838
Stroke history	-0.095	-0.616	0.538
Drinking	0.006	0.099	0.921
BMI	-0.001	-0.042	0.966
Heart Rate		-	
<60	0.151	2.000	0.047
60-79	Ref	-	-
≥80	0.049	0.792	0.429
Diabetes	0.143	2.783	0.006
Aspirin	0.118	1.486	0.139
ADP receptor inhibitor	-0.012	-0.226	0.822
Statins	0.042	0.663	0.508

Ln-transformed EMP was the dependent variable. Age, heart rate, BMI, and SBP were transformed into categorical variables: age: ≤50, 51-60, 61-70, ≥71 years; heart rate: <60, 60-79, ≥80 bpm; BMI: 18.5-23.99, 24-27.99, ≥28 kg/m^2.

ln EMP = 0.151×Heart rate (heart rate < 60 bpm) + 0.143×Diabetes + 2.879.

Definition of abbreviations: CHD, coronary heart disease; BMI, body mass index.

Supplementary Table 3. Multiple regression report in diabetic CHD group: EMPs as the dependent variable.

**Independent variable**	**β**	***t***	***P***
Constant	3.193	18.060	<0.001
Sex	0.079	0.953	0.343
Age	-0.077	-1.557	0.123
Hypertension	0.121	1.395	0.167
Hyperlipidemia	0.031	0.332	0.741
Myocardial infarction history	-0.078	-0.279	0.781
Stroke history	-0.067	-0.363	0.718
Drinking	0.063	0.688	0.493
BMI	0.050	0.940	0.350
Heart Rate			
<60	0.241	2.122	0.037
60-79	ref	-	-
≥80	-0.030	-0.309	0.758
Aspirin	0.152	1.215	0.228
ADP receptor inhibitor	-0.088	-1.032	0.305
Statins	-0.019	-0.191	0.849

Ln-transformed EMP was the dependent variable. Age, heart rate, BMI, and SBP were transformed into categorical variables: age: ≤50, 51-60, 61-70, ≥71 years; heart rate: <60, 60-79, ≥80 bpm; SBP: <120, 120-139, 140-159, ≥160 mmHg; BMI: 18.5-23.99, 24-27.99, ≥28 kg/m^2.

ln EMP = 0.241×Heart rate (Heart rate < 60 bpm) + 3.193.

Definition of abbreviations: CHD, coronary heart disease; BMI, body mass index.

Supplementary Table 4. Multiple regression report in non-diabetic CHD group: EMPs as the dependent variable.

Independent variable	β	*t*	*P*
Constant	3.050	22.717	<0.001
Sex	-0.069	-0.911	0.364
Age	0.023	0.679	0.498
Hypertension	0.085	1.309	0.193
Hyperlipidemia	0.106	1.139	0.257
Stroke history	-0.004	-0.012	0.991
Drinking	-0.041	-0.566	0.573
BMI	-0.030	-0.638	0.525
Heart Rate			
<60	0.024	0.235	0.815
60-79	Ref	-	-
≥80	0.126	1.521	0.131
Aspirin	0.016	0.146	0.884
ADP receptor inhibitor	0.055	0.819	0.415
Statins	0.107	1.294	0.198

Ln-transformed EMP was the dependent variable. Age, heart rate, BMI, and SBP were transformed into categorical variables: age: ≤50, 51-60, 61-70, ≥71 years; heart rate: <60, 60-79, ≥80 bpm; BMI: 18.5-23.99, 24-27.99, ≥28 kg/m^2.
Definition of abbreviations: CHD, coronary heart disease; BMI, body mass index.

Supplementary Table 5. Multiple linear stepwise regression: subgroup analysis by hypertension, hyperlipidemia, and application of CCB.

Independent variable	Standardized β	t	P
Hypertension			
BMI	0.843	20.197	<0.001
Diabetes	0.136	3.248	0.001
Non-Hypertension			
BMI	0.488	2.681	0.009
Heart rate	0.406	2.201	0.030
Diabetes	0.069	1.461	0.147
Hyperlipidemia			
BMI	0.954	13.189	<0.001
Diabetes	-0.011	-0.159	0.874
Non-Hyperlipidemia			
BMI	0.309	2.065	0.040
Diabetes	0.129	3.85	<0.001
Age	0.283	2.065	0.040
Heart rate	0.268	1.995	0.048
With CCB			
BMI	0.826	11.301	<0.001
Diabetes	0.124	1.698	0.094
Without CCB			
BMI	0.566	4.593	<0.001
Diabetes	0.102	3.054	0.003
Age	0.321	2.572	0.011

Definition of abbreviations: CCB, calcium channel blockers; BMI, body mass index.
